# Dr. Helen De Witt Justin

**Published:** 1914-09

**Authors:** 


Dr. Helen De Witt Justin, Woman's Medical College of N. Y. City, 1879, died July 19, shortly after a serious operation at Rochester, Minn. She was buried at Rochester, N. Y. She was born in 1853 at Belleville, N. J., but was educated at Norwich, Conn., and had lived in Europe for four years before beginning her medical studies· She was the valedictorian of her medical class. In 1880, she married Dr. Joel Gilbert Justin, then of the faculty of the Syracuse University Medical School. They moved to Rochester in 1898 and later to Castile, N. Y., serving in the Greene Sanitarium. Her husband died in 1911.

